# Adverse drug events and the associated factors in patients with chronic Chagas disease

**DOI:** 10.1590/0037-8682-0443-2019

**Published:** 2020-04-22

**Authors:** Luiza Braz da Cunha Lopes, Raquel Rodrigues Pereira, Patricia Mello Andrade, Fernanda Martins Carneiro, Mauro Felippe Felix Mediano, Sophia Isabel Linnemann Kilgore, Alejandro Marcel Hasslocher-Moreno, Andréa Silvestre de Sousa, Manoel Marques Evangelista Oliveira, Roberto Magalhães Saraiva, Marcelo Teixeira de Holanda, Gilberto Marcelo Sperandio da Silva

**Affiliations:** 1 Fundação Oswaldo Cruz, Instituto Nacional de Infectologia Evandro Chagas, Rio de Janeiro, RJ, Brasil.; 2 Fundação Oswaldo Cruz, Instituto Oswaldo Cruz, Rio de Janeiro, RJ, Brasil.

**Keywords:** Chagas disease, Adverse drug event, Cardiomyopathy

## Abstract

**INTRODUCTION:**

Herein, we aimed to identify the factors associated with adverse drug events (ADEs) in chronic Chagas disease (CD) patients.

**METHODS:**

We analyzed 320 medical notes from 295 patients. The Naranjo algorithm was applied to determine the cause of ADEs. Mixed effects logistic regression was performed to evaluate the factors associated with ADEs.

**RESULTS:**

ADEs were described in 102 medical notes (31.9%). Captopril was most frequently associated with ADEs. Age (RR 0.96; 95%CI 0.94-0.99) and cardiac C/D stages (RR 3.24; 95%CI 1.30-4.58) were the most important clinical factors associated with ADEs.

**CONCLUSIONS:**

Close follow-up is warranted for CD patients.

Chagas disease (CD) is a public health problem that affects approximately 8 million people worldwide^1^. Most individuals in the chronic phase of the disease present the indeterminate clinical form, which is characterized by the absence of clinical, radiological, and electrocardiographic manifestations of cardiac or digestive involvement^2^ ^,^ ^3^. Approximately 30% of the patients with chronic Chagas disease will develop cardiac and/or digestive symptoms and may require symptomatic pharmacological treatment^2^ ^,^ ^3^. Many patients also present comorbidities such as arterial hypertension, diabetes mellitus, and dyslipidemia, which are conditions that demand the use of one or more drugs for treatment^4^. However, these drugs may interact and contribute to the occurrence of adverse drug events (ADEs) that might not be related to the therapeutic dose. Therefore, the present study aimed to identify factors that might be associated with ADEs among patients with chronic CD.

This retrospective cohort study consisted of patients with chronic CD from Evandro Chagas National Institute of Infectious Diseases (INI) of Oswaldo Cruz Foundation (Fiocruz). The diagnosis of CD was confirmed by two different serological tests (ELISA and indirect immunofluorescence assay) for all patients that visited our outpatient clinical service between 1986 and 2015. The present study was approved by the Evandro Chagas National Institute of Infectious Disease Review Board (number 09935312.0.0000.5262). 

The clinical and sociodemographic variables included in the present study were age, sex, level of education, race, marital status, alcohol consumption, stage of Chagas heart disease (A, B1, B2, C, and D)^2^, number of drugs per prescription, causality, and severity of ADEs. The algorithm of Naranjo was used to determine the causality of the ADE. This algorithm considers the time compatibility between the start of reaction and drug use, the nature of the event and its pharmacological characteristics, and medical or pharmacological plausibility. The algorithm consists of ten questions, with two choices, positive ("yes") or negative ("no"). Based on the scores generated by the algorithm, the ADEs were classified as doubtful (score 0), possible (score 1-4), likely (score 5-8), or defined (score ≥ 9)^5^. The drugs suspected of causing ADEs were identified. The primary outcome was the presence of clinically relevant ADEs for patients who had their drug prescription changed and presented a causality equal to or greater than 1^5^ ^,^ ^6^. The reaction was also described in the leaflet of the suspected drug. ADEs were classified into four categories of intensity: 1) mild, usually transient, requiring no special treatment and not interfering with the volunteer's normal daily activities; 2) moderate, reaction that altered the normal activity of the patient, thereby warranting hospital or emergency care services and withdrawal from work; 3) severe, a reaction that directly threatened the patient's life, and 4) fatal, a reaction that led to the patient's death^6^ ^,^ ^7^.

Statistical analyses were performed using RStudio (RStudio Team, 2015). Descriptive analysis was expressed as mean (SD). The mixed effects logistic regression was performed using glmer function in the lme4 R package and estimates of the risk ratios were calculated using odds_to_rr function in the sjstats package. Models included a random intercept by patient. Multivariate analysis included age, sex, stages of CD, and number of prescribed drugs that were selected due to their association with the ADEs described in the literature. The significance level was set at 5%.

We analyzed 320 medical notes from 295 patients (mean age, 57.4 years) followed from November 1986 to December 2015 to measure the frequency of ADEs. Stages C and D were grouped together owing to the small number of cases (48 with stage C and 4 with stage D). Of the 102 ADEs, 77 patients had at least one ADE and 25 had more than one ADE (Table 1). 

**TABLE 1:** Characteristics of the study population and the association estimates (risk ratio) for adverse drugs events (n=320 medical notes from 295 patients).

Variable	n (320)	%	Univariate RR	Multivariate RR
			(95%CI)*	(95%CI)*
**Age**				
Mean(SD)	57.4	---	0.96	0.96
	(11.2)		(0.94-0.99)	(0.94-0.99)
**Sex**				
Men	131	40.9	Reference	---
Women	189	59.1	0.99	1.23
			(0.50-1.66)	(0.64-1.94)
**Schooling**				
Illiterate	48	15	Reference	---
Primary	219	68.4	1.14	---
			(0.42-2.19)	
Secondary	47	14.7	0.73	---
			(0.15-2.09)	
Tertiary	6	1.9	0.57	---
			(0.01-3.06)	
**Race**				
White	143	44.7	Reference	---
Mulatto	127	39.7	1.01	---
			(0.48-1.77)	
Black	50	15.6	1.11	---
			(0.40-2.15)	
**Marital status**				
Single	111	34.7	Reference	---
Married	186	58.1	1.21	---
			(0.62-1.97)	
Widowed	23	7.2	0.29	---
			(0.03-1.56)	
**Alcohol Consumption**				
No	133	66.8	Reference	---
Yes	66	33.2	0.98	---
			(0.45-1.76)	
**Stages of Chagas disease**				
Indeterminate	51	15.9	Reference	Reference
A	128	40.0	1.43	1.56
			(0.45-3.11)	(0.49-3.28)
B1	67	20.9	1.87	2.00
			(0.57-3.71)	(0.61-3.84)
B2	22	6.9	3.36	3.27
			(1.08-4.75)	(0.96-4.75)
C	48	15.0	3.11	3.24
			(1.24-4.51)	(1.30-4.58)
D	4	1.2		
**Number of drugs per prescription**				
0-1	9	2.8	Reference	---
2-4	103	32.2	1.55	1.35
			(0.14-4.01)	(0.12-3.88)
5-12	208	65.0	1.20	1.01
			(0.10-3.79)	(0.08-3.64)
**Causality**				
Definite	41	12.8	(< 0.001)**	---
Probable	24	7.5		---
Possible	37	11.6		---
Doubtful/no event	218	68.1		---
**Severity**				
Mild	59	18.4	(< 0.001)***	---
Moderate	38	11.9		---
Severe	5	1.6		---
No event	218	68.1		---

*Mixed effects logistic regression; **Pearson's Chi-squared test; ***Fisher's exact test.

The variables that maintained significant associations with ADEs in the multivariate analysis were age (RR 0.96; 95%CI 0.94-0.99) and the C/D stages of Chagas heart disease (RR 3.24; 95%CI 1.30-4.58) (Table 1). 

The angiotensin converting enzyme inhibitor (ACE inhibitor) (n = 34; 33.3%) was the most frequent cause of ADEs. This drug was followed by amiodarone (n = 13; 12.7%), acetylsalicylic acid (ASA) (n = 9; 8.8%), hydrochlorothiazide (7; 6.9%), spironolactone (n = 6; 5.9%), and warfarin (n = 5; 4.9%) (Table 2).

**TABLE 2:** Drugs that resulted in ADEs in the study population.

Drug name	n (%)
ACE inhibitors*	34 (33.3)
Amiodarone	13 (12.7)
Acetylsalicylic acid	9 (8.8)
Hydrochlorothiazide	7 (6.9)
Spironolactone	6 (5.9)
Warfarin	5 (4.9)
Digoxin	4 (3.9)
Propranolol	4 (3.9)
Isosorbide	4 (3.9)
Amlodipine	3 (2.9)
Benznidazole	3 (2.9)
Carvedilol	2 (2.0)
Furosemide	2 (2.0)
Diazepam	1 (1.0)
Losartan	1 (1.0)
Methyldopa	1 (1.0)
Nifedipine	1 (1.0)
Sotalol	1 (1.0)
Ferrous sulfate	1 (1.0)
Total number of drugs (n=19)	Total events (n=102)

*Angiotensin-converting enzyme inhibitors (24 captopril and 10 enalapril).

The types of ADEs detected are presented in Table 3. The most frequent ADEs among the study population were cough (n=28; 8.8%) and hyperkalemia (n=3; 0.9%) for the ACE inhibitor; endocrine disorders (n=4; 1.3%) and respiratory disorders (n=3; 0.9%) for amiodarone; and bleeding (n=4; 1.3%) and epigastralgia (n=3; 0.9%) for acetylsalicylic acid.

**TABLE 3:** Frequency of drugs and the distribution of ADEs in the patient medical notes.

Drug	ADRs	Frequency, n (%)	Total, n (%)
**ACE inhibitor (Captopril + enalapril)**	Cough	28 (8.8)	34 (10.6)
	Nausea /Gastric intolerance	1 (0.3)	
	Hypotension	1 (0.3)	
	Hyperkalemia	2 (0.6)	
	Presyncope	1 (0.3)	
	Dizziness	1 (0.3)	
**Amiodarone**	Endocrine disorders	4 (1.3)	13 (4.1)
	Dizziness	1 (0.3)	
	Cerebellar syndrome	1 (0.3)	
	Drowsiness and hypotension	1 (0.3)	
	Ophthalmic disorders	2 (0.6)	
	Hepatitis	1 (0.3)	
	Respiratory disorders	3 (0.9)	
**Acetylsalicylic acid**	Bleeding	4 (1.3)	9 (2.8)
	Epigastralgia	3 (0.9)	
	Urticaria	2 (0.6)	
**Hydrochlorothiazide**	Nausea and epigastric pain	1 (0.3)	7 (2.2)
	Hypokalemia	1 (0.3)	
	Hyponatremia	1 (0.3)	
	Renal disorders	1 (0.3)	
	Hypotension	2 (0.6)	
	Orthostatic hypotension	1 (0.3)	
**Spironolactone**	Hyperkalemia	3 (0.9)	6 (1.9)
	Gynecomastia	2 (0.6)	
	Hypotension	1 (0.3)	
**Warfarin**	Increased INR	1 (0.3)	5 (1.6)
	Bleeding	1 (0.3)	
	Hematuria	1 (0.3)	
	Dermatitis	2 (0.6)	
**Digoxin**	Gynecomastia	1 (0.3)	4 (1.3)
	Bradycardia	1 (0.3)	
	Weakness	1 (0.3)	
	Bigeminy	1 (0.3)	
**Propranolol**	Hypotension	1 (0.3)	4 (1.3)
	Intense bradycardia	1 (0.3)	
	Bronchospasm	1 (0.3)	
	Dizziness	1 (0.3)	
**Isosorbide**	Hypotension	1 (0.3)	4 (1.3)
	Severe headache	1 (0.3)	
	Headache with nitrate	1 (0.3)	
	Dysphagia and dizziness	1 (0.3)	
**Amlodipine**	MMII Edema	1 (0.3)	3 (0.9)
	Edema	1 (0.3)	
	Lower limb edema	1 (0.3)	
**Benznidazole**	Leukopenia	1 (0.3)	3 (0.9)
	Memory deficit	1 (0.3)	
	Amnesia and mental confusion	1 (0.3)	
**Carvedilol**	Bronchospasm	1 (0.3)	2 (0.6)
	Erectile dysfunction	1 (0.3)	
**Furosemide**	Paresthesia	1 (0.3)	2 (0.6)
	Hypotension	1 (0.3)	
**Diazepam**	Discouragement and daytime prostatitis	1 (0.3)	1 (0.3)
**Losartan**	Dizziness and drowsiness	1 (0.3)	1 (0.3)
**Methyldopa**	Postural hypotension	1 (0.3)	1 (0.3)
**Nifedipine**	Hypotension and redness on face	1 (0.3)	1 (0.3)
**Sotalol**	Bradycardia, dizziness, and sweating	1 (0.3)	1 (0.3)
**Ferrous sulfate**	Dizziness	1 (0.3)	1 (0.3)

The ACE inhibitor is frequently used to treat patients with chronic heart failure; however, some patients experience intolerance, mainly with captopril and enalapril, that may cause dry cough^8^. In the present study, 28 (8.8%) cases of cough were observed and classified as adverse reactions related to captopril use, a finding that is very similar to that of Hamad et al*.* ^9^ (5%). Although amiodarone is considered safe for the treatment of arrhythmias, it can cause thyroid dysfunction, which should be monitored at baseline and every 6 months during therapy^2^ ^,^ ^8^ ^,^ ^10^. Silva et al*.* ^11^ reported that 56.9% of patients had thyroid hormone alterations when treated with amiodarone. In the present study, amiodarone was discontinued due to hyperthyroidism in four (1.3% of events) patients. Thus, the low-frequency events could be consequence of the close and regular multidisciplinary follow-up conducted with our patients. Four minor gastrointestinal bleedings (1.3% of total ADRs) were also observed in our study, thereby leading to the discontinuation of ASA treatment. Such finding aligns with that of the literature as low-dose ASA was reported to be associated with the increased risk of any bleeding^12^. Herein, we also observed hyperkalemia due to spironolactone. Three hyperkalemia events (0.9%) occurred, a finding similar to Adorisio^8^ and Botoni et al^13^ who revealed that 4.8% of patients administered spironolactone presented hyperkalemia. 

In the present study, the worsening of clinical status may have led to an increase in the number of drugs and consequently, the frequency of ADEs, as previously demonstrated^14^. However, although some studies have demonstrated that older patients are at a higher risk of developing ADEs, such finding was not confirmed herein. Instead, lower age was associated with more severe clinical presentations (B2, C and D; mean age of 54.1 years) in the present study. Patients with stages C/D had increased risk of ADEs compared to indeterminate patients. The increase in cardiac damage may lead to a greater risk of ADEs. For the severity classification, most ADEs were mild; this was followed by moderate reactions. Only five ADEs were identified as severe. The use of enalapril to replace captopril in patients requiring the use of ACE inhibitors may be better indicated for the prevention of ADEs and better adherence to treatment^2^. However, when cough is recurrent, the use of other ARBs, such as losartan, may serve as an alternative treatment^2^. Through this study, we could observe the frequency and type of ADEs caused by symptomatic or non-symptomatic pharmacological treatments in patients with chronic CD, as well as factors and drugs associated with these events. A close follow-up of these patients may thus reduce the frequency of ADEs. Recent clinical trials performed in this setting by our research group have confirmed this hypothesis in patients with heart failure induced by chronic CD^15^. 

In conclusion, the advanced stages of Chagas heart disease (C/D) and age have a significant association with the presence of ADEs. Furthermore, when cough was identified as the main frequently observed adverse event of the ACE inhibitors, losartan could be administered as an alternative to minimize this side effect.

## References

[1] World Health Organization (2019). Chagas disease (American trypanosomiasis).

[2] Dias JCP, Jr. AN Ramos, Gontijo ED, Luquetti A, Shikanai-Yasuda MA, Coura JR (2016). Brazilian Consensus on Chagas Disease, 2015. Epidemiol E Serviços Saúde.

[3] Rassi A, Rassi A, Marin-Neto JA (2010). Chagas disease. Lancet.

[4] Vizzoni AG, Varela MC, Sangenis LHC, Hasslocher-Moreno AM, do Brasil PEAA, Saraiva RM (2018). Ageing with Chagas disease: an overview of an urban Brazilian cohort in Rio de Janeiro. Parasit Vectors.

[5] Naranjo CA, Busto U, Sellers EM, Sandor P, Ruiz I, Roberts EA (1981). A method for estimating the probability of adverse drug reactions. Clin Pharmacol Ther.

[6] Hasslocher-Moreno AM, Brasil PEA do A, Sousa AS de, Xavier SS, Chambela MC, Silva GMS da (2012). Safety of benznidazole use in the treatment of chronic Chagas disease. J Antimicrob Chemother.

[7] Silva GMS da, Mediano MFF, Hasslocher-Moreno AM, Holanda MT de, Silvestre AS, Sangenis LHC (2017). Benznidazole treatment safety: the Médecins Sans Frontières experience in a large cohort of Bolivian patients with Chagas’ disease. J Antimicrob Chemother.

[8] Adorisio R, Luca L de, Rossi J, Gheorghiade M (2006). Pharmacological treatment of chronic heart failure. Heart Fail Rev.

[9] Hamad E, Mather PJ, Srinivasan S, Rubin S, Whellan DJ, Feldman AM (2007). Pharmacologic therapy of chronic heart failure. Am J Cardiovasc Drugs Drugs Devices Interv.

[10] Ribeiro AL, Nunes MP, Teixeira MM, Rocha MOC (2012). Diagnosis and management of Chagas disease and cardiomyopathy. Nat Rev Cardiol.

[11] Silva JR, Guariento ME, Fernandes GA, Maciel RMB, Ward LS (2004). Impact of long-term administration of amiodarone on the thyroid function of patients with Chagas’ disease. Thyroid Off J Am Thyroid.

[12] Davies EA, O’Mahony MS (2015). Adverse drug reactions in special populations - the elderly. Br J Clin Pharmacol.

[13] Botoni FA, Poole-Wilson PA, Ribeiro AL, Okonko DO, Oliveira BM, Pinto AS (2007). A randomized trial of carvedilol after renin-angiotensin system inhibition in chronic Chagas cardiomyopathy. Am Heart J.

[14] Carmona-Torres JM, Cobo-Cuenca AI, Recio-Andrade B, Laredo-Aguilera JA, Martins MM, Rodríguez-Borrego MA (2018). Prevalence and factors associated with polypharmacy in the older people: 2006-2014. J Clin Nurs.

[15] Chambela MDC, Mediano MFF, Carneiro FM, Ferreira RR, Waghabi MC, Mendes VG (2020). Impact of pharmaceutical care on the quality of life of patients with heart failure due to chronic Chagas disease: Randomized clinical trial. Br J Clin Pharmacol.

